# Autism spectrum disorders pathogenesis: Toward a comprehensive model based on neuroanatomic and neurodevelopment considerations

**DOI:** 10.3389/fnins.2022.988735

**Published:** 2022-11-03

**Authors:** Athanasios Beopoulos, Manuel Géa, Alessio Fasano, François Iris

**Affiliations:** ^1^Bio-Modeling Systems, Paris, France; ^2^Division of Pediatric Gastroenterology and Nutrition, Mucosal Immunology and Biology Research Center, Massachusetts General Hospital for Children, Boston, MA, United States; ^3^Division of Pediatric Gastroenterology and Nutrition, Center for Celiac Research and Treatment, Massachusetts General Hospital for Children, Boston, MA, United States

**Keywords:** autism spectrum disorder (ASD), autonomic nervous system (ANS), neurodevelopment, serotonin, RNA editing, maternal inflammation, kynurenine metabolites, synaptic pruning

## Abstract

Autism spectrum disorder (ASD) involves alterations in neural connectivity affecting cortical network organization and excitation to inhibition ratio. It is characterized by an early increase in brain volume mediated by abnormal cortical overgrowth patterns and by increases in size, spine density, and neuron population in the amygdala and surrounding nuclei. Neuronal expansion is followed by a rapid decline from adolescence to middle age. Since no known neurobiological mechanism in human postnatal life is capable of generating large excesses of frontocortical neurons, this likely occurs due to a dysregulation of layer formation and layer-specific neuronal migration during key early stages of prenatal cerebral cortex development. This leads to the dysregulation of post-natal synaptic pruning and results in a huge variety of forms and degrees of signal-over-noise discrimination losses, accounting for ASD clinical heterogeneities, including autonomic nervous system abnormalities and comorbidities. We postulate that sudden changes in environmental conditions linked to serotonin/kynurenine supply to the developing fetus, throughout the critical GW7 – GW20 (Gestational Week) developmental window, are likely to promote ASD pathogenesis during fetal brain development. This appears to be driven by discrete alterations in differentiation and patterning mechanisms arising from *in utero* RNA editing, favoring vulnerability outcomes over plasticity outcomes. This paper attempts to provide a comprehensive model of the pathogenesis and progression of ASD neurodevelopmental disorders.

## Introduction

Autism is not a single disorder, but a spectrum of related disorders with a shared core of symptoms defined by deficits in communication, social reciprocity and repetitive, stereotypic behaviors. While previously thought to present an extreme sex/gender bias (four times more common among males than females) it is now established that this apparent bias is largely due to affected females being more capable of disguising their condition than affected males (Dworzynski et al., 2012; Kreiser and White, 2014; Mandic-Maravic et al., 2015; Hull et al., 2017; Ratto et al., 2018; Young et al., 2018). Currently, autism spectrum disorders (ASD) affects around 1 in 68 children around the world, a 35-fold increase since the earliest epidemiologic studies were conducted in the late 1960s and early 1970s. However, this sharp increase may be also related- to some extent- to diagnostic, specificity, sensitivity or administrative biases (Monteiro et al., 2015; Randall et al., 2018; Ghandour et al., 2019). ASD is characterized by early increases in brain volume and cortical thickness during infancy and the toddler years (2–4 years) in young autistic males and females, followed by an accelerated rate of decline in size, and perhaps degeneration, from adolescence to late middle age in this disorder (Hazlett et al., 2017).

In most cases, this aberrant brain growth does not occur at birth, but rather develops throughout the first 2 years of life after birth. Early post-natal brain overgrowth is then followed by arrest of growth. The sites of regional overgrowth in ASD include frontal and temporal cortices and the amygdala and, in some regions, and individuals, growth arrest may be followed by degeneration (Courchesne et al., 2007). Brain overgrowth follows an important gradient in the cerebrum: greatest in the frontal and temporal cortices, which are most abnormally enlarged, and least in the occipital cortex, with young autistic males presenting a very significant excess of neuron number in the dorsolateral prefrontal cortex (PFC) (Courchesne et al., 2011). Since there is no known neurobiological mechanism in humans capable of generating during postnatal life the large excesses of frontal cortical neurons, the great magnitude of this excess is most probably due to dysregulation of layer formation and layer-specific neuronal differentiation at prenatal developmental stages, rather than to any known postnatal event or mechanism. Therefore, the peak period for detecting and studying the early biological basis of autism is from prenatal life to the first three years postnatally.

Some seasonal cyclicity appears associated with the percentage of children affected by ASD, intellectual disabilities, and learning limitations, with rates being greatest for children conceived in the winter months, and lowest for those conceived during the summer (Mackay et al., 2016). Conception in the winter season is associated with a 6% increased risk of ASD presentation as compared with summer (Zerbo et al., 2011).

Many individuals with ASD have symptoms of associated co-morbidities, including seizures, sleep problems, metabolic conditions, and gastrointestinal (GI) disorders, which have significant health, developmental, social, and educational impacts. The neuroanatomical and biochemical characteristics that have been associated with autism pathogenesis *in utero* (Rossignol and Frye, 2012; Stoner et al., 2014; Zielinski et al., 2014) involve mechanisms that are direct consequences of the effects of low-grade, feverless, systemic inflammatory events (Nankova et al., 2014), while the protective mechanisms against autism pathogenesis have strong anti-inflammatory components (Scumpia et al., 2014). The gut microbiome drives immunoregulation (in particular during the first 3 years of life) and faulty immunoregulation, as well as inflammation, predispose to psychiatric disorders, including autism, while psychological stress drives further inflammation via pathways that involve the gut microbiome (Kostic et al., 2015). Thus, while ASD is significantly associated with subsequent incidence of inflammatory bowel disease (Kim et al., 2022), contrary to many psychological and psychiatric diseases there is a lack of evidence for systemic low-grade inflammation in association with ASD affected individuals (Prosperi et al., 2019).

This paper attempts to explore the most plausible mechanisms involved in the pathogenesis of ASDs and to relate them to the phenotypic and symptomatic manifestations of common characteristics. As our hypothesis paper covers a wide range of different topics and disciplines, we have often tried to include a general description of the mechanisms involved in neurotypical brain development and their respective components (immune regulation, kynurenine pathway, mRNA editing, functional communication between brain areas, etc.) in order to facilitate reading by different specialties and a wider audience.

## Materials and methods

The authors conducted an in-depth review of the literature and used a systems biology approach to integrate the complex mechanisms of fetal brain development to then decipher the neurodevelopmental origins encountered in individuals with ASD. Throughout our literature review, we strongly favored studies on human subjects, and in the few cases where this is not the case, it is clearly stated in the text. The analytical procedure implemented (CADI™ : Computer-Assisted Deductive Integration) associates algorithmics and heuristics. The logic behind this model-building approach does not assume functional linearity within biological systems and the components of a model do not incorporate solely what is known. Indeed, since this approach relies upon strict and systematic implementation of negative selection of hypotheses, models arising from this procedure contain elements that have never been described but cannot be refuted by current knowledge and/or available biological data, thereby generating novel understanding. This model-building approach has proven its efficacy in a number of biological research domains, including the discovery of hitherto unsuspected biological mechanisms, pathways, and interactions directly associated with phenotypic transitions *in vivo* (be they pathological or developmental) (Gadal et al., 2003, 2005; Iris et al., 2009; Pouillot et al., 2010; Turck and Iris, 2011; Iris, 2012; Nussbaumer et al., 2016). CADI™ modeling has led to discoveries and patents in the fields of infectious diseases, oncology, neurology, psychiatry, dermatology, immunology, metabolic disorders, innovative bioprocesses for industrial biotech and the creation of new companies exploiting these patents. CADI™ models describe the biological phenomena involved in pathological states and provide novel mechanistic integrations to explain the cause of certain diseases, identify and select predictive biomarkers, and offer new combinations of molecules and new therapeutic strategies. Further information on the Computer-Assisted Deductive Integration method can be found in Iris et al. (2018).

## Analysis

### The neuro-anatomical specificities of autism

In view of the enormous heterogeneity that characterizes the spectrum of autism, neuroanatomic variations are to be expected. One of the most common disparities in ASD development involves the amygdala, located deep and medially within the temporal lobes of the cerebrum. In typical fetal brain development, the amygdala displays structural connectivity across the cortex, particularly toward frontal and temporal lobes, by gestational week GW13 (Vasung et al., 2010) and a mature structure by 8 months (Vasung et al., 2010). Columns of neuroblasts from the inferior germinal eminence (GE), located ventral to the developing amygdala (Ulfig et al., 2000), migrate along radial glial fibers initiating amygdala development (Müller and O’Rahilly, 1990; Ulfig et al., 2003). As fetal development progresses, the GE shrinks to a small population of cells that surrounds the ventral section of the maturing amygdala. In humans, the paralaminar nucleus (PL) begins to develop during the 8th and 9th gestational month, residing and maturing at the location of the remnant GE (Ulfig et al., 2003), while mature PL still contains subpopulations of immature neurons (Bernier et al., 2000; Yachnis et al., 2000; Fudge and Tucker, 2009; Zhang et al., 2009). PL is a unique subregion of the amygdala, densely innervated by serotonergic fibers with high numbers of receptors for corticotropin releasing hormone and benzodiazepines. It is thought to play key roles in the normal growth and function of the amygdala, especially due to the resident immature cells that appear to confer enhanced neuronal plasticity (deCampo and Fudge, 2012).

Of all the various structural brain abnormalities reported in autism and related to the amygdala (reviewed in Donovan and Basson, 2017), the most consistent seem to be:

- rapid and early increases in the size of the right and left amygdala, which correlate positively with the extent of their deficits in social interaction and communication at age 5 (Ortiz-Mantilla et al., 2010).

- greater amygdala spine density in youths with ASD than in age-matched controls with typical development (TD) (<18 years), but which decreases as they grow older, a pattern not found in TD (Weir et al., 2018).

- initial overabundance of amygdala neurons in young ASD subjects, followed by a reduction in all nuclei during adult years, whereas in TD, there is an increase of mature neurons in the basal and accessory basal nuclei from child to adult, concurrently with a decrease of immature neurons in the PL (Avino et al., 2018).

This is suggestive of a sustained contribution of mature neurons from the PL nucleus toward other nuclei of the neurotypic amygdala, and that this developmental path may be altered in ASD, which may explain the changes in volume seen in ASD and other neurodevelopmental or neuropsychiatric disorders.

The amygdala, stretching from 1 to 4 cm with an average of about 1.8 cm, has a wide range of connections with the brain. The hippocampus, entorhinal cortex, basal ganglia (particularly the striatum), brainstem, thalamus, and hypothalamus are all part of this organization. It is also linked to the limbic system, the associative cortex, the PFC (which controls behavior), the basal forebrain, and other areas. As a result, its stimulation is expected to have a significant impact on the entire brain.

**FIGURE 1:**
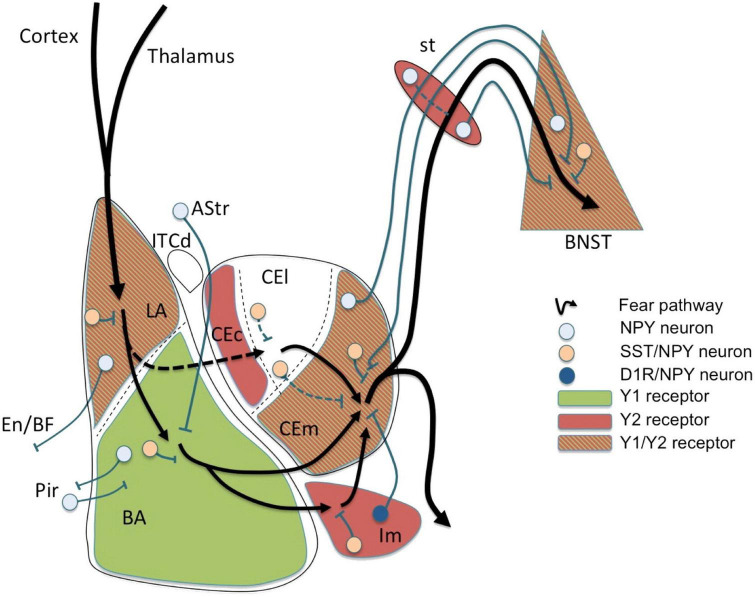
Fear circuit in the amygdala. Cortical and thalamic projections conveying somatosensory (US) and auditory (CS) information are targeting the lateral amygdala (LA), a major location of initial synaptic plasticity. Somatostatin (SST)/neuropeptide Y (NPY)/γ-aminobutyric acid (GABA) neurons may inhibit pyramidal neurons by activation of postsynaptic Y1 and presynaptic Y2 receptors reducing CS-related activation and fear learning. Similarly, SST/NPY neurons in the basolateral nucleus (BLA) may have additional Y1 receptor-dependent inhibiting effects. The role of NPYergic afferent and efferent projections of the basolateral complex (LA and BLA) is not yet clear. Fear related stimuli reach the central amygdala (CEA) also via mediodorsal intercalated neurons (ITCd) to the centrolateral subdivision (CEl) or directly via projections from the BLA to the centromedial subdivision (CEm). NPY neurons may modulate afferent and efferent signals locally in the CEl or via inhibitory connections between CEl and CEm, probably by Y2 receptors. However, the main processing occurs in the CEm (expressing Y1 and Y2 receptors) by (1) local inhibitory SST/NPY/GABA neurons, (2) by NPY/GABA afferents originating from the BNST, and (3) by NPY axon terminals originating from neurons of the main intercalated nucleus (Im). The latter projection consisting of NPY/dopamine receptor D1 (D1R)/GABA neurons in the Im is considered to play an essential role in fear extinction by providing a marked increase in feed-forward inhibition from the BLA to the CEm. Lastly, efferents from the CEm may be further modulated by NPY along the stria terminalis (st) or even in the bed nucleus of the stria terminalis (BNST), both by local interneurons and by bi-directional projections connecting the CEm with the BNST. AStr, amygdala-striatal transition zone; En, entorhinal cortex; Pir, piriform cortex; BF, basal forebrain; CEc, capsular subdivision of the central amygdala nucleus. Figure from Tasan et al. (2016), obtained by Elsevier (No. 5410821023270) under a creative commons license 3&4.

This can be exemplified in one of the amygdala’s major functions in humans which is to regulate emotions, particularly fear and anxiety (Figure 1), and the behaviors that follow from them. In addition, the amygdala has some memory function toward conditioned learning, which is the remembrance of situations that cause fear and anxiety. Thus, the emotion can be reproduced by stimuli or conditions that are similar to those that caused the initial dread and anxiety.

However, ASD-associated neuro-anatomical alterations aren’t limited to the amygdala, and significantly affect the cortex, comprising progressively the six-layer neocortex (I–VI, with the outermost layer I neighboring the pia mater and the innermost layer VI neighboring the white matter), the 5–6 layer mesocortex (lacks or has poorly developed layer IV) and finally the three-layer allocortex. The neocortex takes up 90% of the cortex and mostly involves sensory, motor and associative functions. The mesocortical and allocortical areas form a closed ring at the edge of the hemisphere, forming the limbic areas which are found in the frontal, temporal, parietal, and occipital lobes as well as in the insula (García-Cabezas et al., 2022). Functions of the neocortex rely on a complex architecture of neuronal networks that is key in the excitation/inhibition imbalance observed in several neurodevelopmental disorders, including ASD. The superficial layer 1 (L1), which is consistently affected in autism, mostly comprises local and long-range inputs and sparse inhibitory interneurons that collectively govern brain activities. Its stereotypical organization, generated in early development, where axonal projections synapse onto the dendritic tufts of pyramidal neurons, functions as a crucial integration hub that provides context for sensory information (i.e., local, thalamocortical, corticocortical, and long-range neuromodulation). As it receives connections from feedback and neuromodulatory pathways during its prolonged development, it plays a crucial role in the development, maturation, and function of the whole cortex. Its maturation continues postnatally with changes related to network affinity refinement affecting the whole cortex. Furthermore, interneurons found in Layer I facilitate disinhibition and thus contribute to plasticity and learning. During its development, it transiently accommodates Cajal-Retzius (CR) cells that regulate neuronal networks formation and migrating interneurons (*see below*). The organization of myelin in the cortex is highly diverse, with the content of intracortical myelin increasing progressively in parallel to laminar architecture elaboration from mesocortical agranular areas to koniocortical areas (García-Cabezas et al., 2020). Myelin sheath densities are representative of the extraordinary spatiotemporal organization of cortical networks (Trutzer et al., 2019; Call and Bergles, 2021; Genescu and Garel, 2021).

The most consistent cortical architecture abnormalities related to ASD appear to be:

- Abnormal patterns of overgrowth (7% increase in cerebrum size, with 5 and 10% increase in total gray and white matters, respectively) beginning before the second year, followed by reduced growth and arrested development at the end of childhood persisting into adolescence (Donovan and Basson, 2017);

- Sites of regional overgrowth that include frontal and temporal cortices (Abot et al., 2018);

- Greater spine densities on pyramidal cells in both the superficial and deep cortical layers of the frontal, temporal, and parietal lobe regions (layer II in each cortical location and within layer V of the temporal lobe) as compared to age-matched control subjects (Hutsler and Zhang, 2010);

- Excessive short to medium distance axons with increased branching in the superficial white matter beneath the anterior cingulate cortex (ACC). This finding is consistent with the hypothesis that prefrontal areas are overconnected in autism and probably explains the increased cortical folding in the frontal lobe in autism. In addition, the decrease in myelin thickness in the orbitofrontal cortex (OFC) observed in ASD may indicate altered relationships between prefrontal areas (Zikopoulos and Barbas, 2010). These areas govern emotion, attentional mechanisms and executive control, processes that are severely affected in autism (Barbas, 2000).

- High spine densities, which are associated with decreased brain weights, are most commonly found in ASD subjects with lower levels of cognitive functions (Hutsler and Zhang, 2010);

- Absence of the decline in cortical surface area that is always present in TD between the ages of 9 and 20 (Mensen et al., 2017);

- Irregular, age-related variations in cortical thickness, with a slower rate of thinning from childhood through adolescence and a faster rate of thinning in late adolescence. In TD, on the other hand, thinning occurs during teen years/early puberty (Nunes et al., 2020).

- In females, the volume and surface area of the temporal and frontal gyri and sulci, as well as the surface area of the left superior frontal and right lateral superior temporal gyri, are reduced. However, there is a tendency for the temporal gyri to thicken, resulting in little change in the volume of these areas (Cauvet et al., 2019), and

- In males, increased volume of the left anterior occipital sulcus and reduced thickness of the right para-hippocampal region of the medial occipito-temporal gyrus are related to autistic traits (Cauvet et al., 2019).

Furthermore, cortical networks in layer 1 are disorganized in autism: autistic children have higher variability in the trajectories and thickness of myelinated axons, whereas adults with autism show a decrease in the relative proportion of thin axons. In contrast, neurotypical controls, from children to adults, demonstrate an increase in the density of myelinated axons. Overall, neuron density decreases with age, as does the density of inhibitory interneurons identified with calbindin (CB), calretinin (CR), and parvalbumin (PV). Cortical activity refinement throughout development of cortical networks involved in cognition is likely aided by neurotypical postnatal alterations in layer 1 of the lateral prefrontal cortex (LPFC). This indicates that it is mostly the disruption of feedback pathways maturation, rather than interneurons in layer 1, that plays a crucial role in the development of autism’s excitation-inhibition disequilibrium (Trutzer et al., 2019).

The prominent innervation by serotoninergic neurons and the dense expression of serotonergic receptors in the PFC, which shows overgrowth in ASD children, suggest that serotonin is a major modulator of its function (Wassink et al., 2007; Puig and Gulledge, 2011). Similarly, serotonin prominently promotes amygdala activation in response to emotional stimuli (Bocchio et al., 2016; Sengupta et al., 2017).

It finally should be noted that ASD does not figure among the disorders classified according to the stage of development where cortical elaboration was initially affected [i.e., malformations due to (1) abnormal proliferation/apoptosis; (2) abnormal migration; (3) abnormal late neuronal migration and cortical organization; and (4) cortical development, not otherwise classified; Barkovich et al., 2005]. Rather, ASD has been described as a pathology arising from multiple discrete alterations in brain growth, structure, synaptic organization and modified short- and long-range connectivity in the cortex (Hutsler and Casanova, 2016).

### Fetal brain development

Throughout the following discussion, we have adhered to the officially accepted neuromorphological nomenclature pertaining to fetal brain development. However, interested reader should be made aware of the existence of a new, albeit not yet officially accepted, nomenclature (the Prosomeric Model: Nieuwenhuys and Puelles, 2016; Puelles, 2018, 2021; Watson et al., 2019; Garcia-Calero and Puelles, 2020) with the relation between the classically accepted subdivisions of the neural tube (rhombencephalon, mesencephalon, diencephalon, etc.) and the newly proposed neuromorphological nomenclature being explained in Puelles (2018), Watson et al. (2019), and Garcia-Calero and Puelles (2020).

**FIGURE 2:**
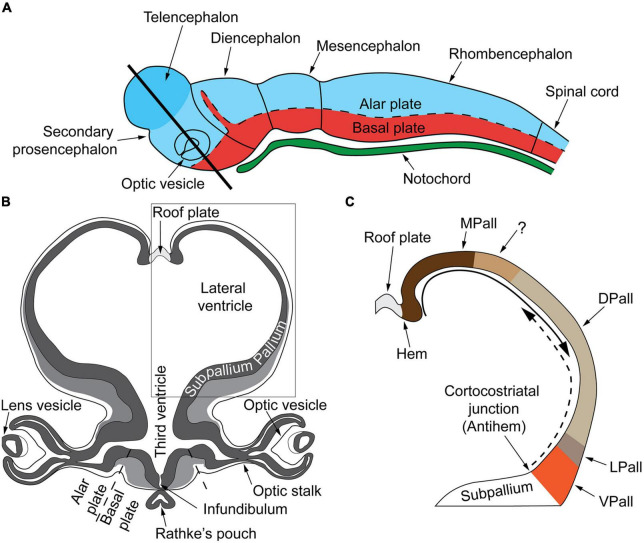
**(A)** Very early cerebral cortex development. Sketch of the mammalian neural tube showing the proneuromeric compartments. The neural tube will develop to form the brain and spinal cord. It is divided to the alar plate (colored in blue) that will later generate neurons associated with sensory functions (somatic and visceral), and the basal plate (red) that will primarily contain motor neurons. The notochord, of mesodermal origin (green) provides directional cues to the surrounding tissues. The telencephalic vesicle (highlighted in darker blue) grows out of the alar plate of the secondary prosencephalon. **(B)** Very early cerebral cortex development. Section through the secondary prosencephalon at the level of the black line in **(A)** shows the optic vesicles and the telencephalic vesicles; the telencephalic vesicles consist of pallial and subpallial territories. **(C)** Two organizers direct the patterning of the pallium: the hem, at the border of the roof plate and the prospective hippocampus, and the antihem, at the corticostriatal junction near the prospective olfactory cortex. The morphogen molecules segregated by these organizers form overlapping gradients (solid and dashed arrows) that pattern the pallium in four sectors: medial, dorsal, lateral, and ventral pallial sectors. The distinction of two parts on the MPall sector corresponding to allocortex (hippocampus) and the adjacent mesocortex (marked with “?”) is hypothesized based on architectonic analysis of adult rats and primates. DPall, dorsal pallium; LPall, lateral pallium; MPall, medial pallium; VPall, ventral pallium. Figure from García-Cabezas and Zikopoulos (2019), obtained by Elsevier (No. 5410821023270) under a creative commons license 3&4.

During embryonic development the ectoderm differentiates to form epithelial and neural tissues. The external (dorsal surface) ectoderm forms the neural plate that folds to generate the neural tube (i.e., the CNS precursor) and the neural crest (multipotent transient lineages that form the paravertebral and prevertebral ganglionic chain and chromaffin tissues). The neural tube generates three primary brain vesicles during GW4–GW5 in human development: the forebrain (prosencephalon) that matures into the cerebrum (telencephalon: cortex and white matter) and the diencephalon (thalamus, hypothalamus, epithalamus, subthalamus, and optical vesicles); the midbrain (mesencephalon: superior and inferior colliculi) and the rhombencephalon that progresses into the metencephalon (the pons and cerebellum) and the myelencephalon (the medulla oblongata). The midbrain and the rhombencephalon (pons, cerebellum, and medulla oblongata) form the brainstem, which begins development around GW6–GW7 and matures in a caudal to rostral arc (Figure 2A).

The dorsoventral axis of the telencephalon is further separated, with dorsal (pallial) and ventral (subpallial) domains giving rise to the cerebral cortex and basal ganglia and amygdala, respectively. Furthermore, morphological regions can be classified into auxiliary progenitor domains (prosomeres) based on gene expression (Figures 2B,C), that overlap with these anatomic subdivisions.

Arousal, respiration, heart rate, and gross motions of the body and head are all mediated by the medulla, and medullary activities appear before those of the pons, which appear before those of the midbrain. As a result, by GW7–GW9, the fetus is moving spontaneously, starts to “breathe,” and by GW25 demonstrates stimulus-induced heart rate accelerations. As the pons, that mediates arousal, body movements, vestibular and vibroacoustic perceptions, takes longer to mature, the fetus only starts responding to vibroacoustic and loud noises coming from the exterior environment with arousal and body movements from GW20 to GW27.

The inferior-auditory colliculus begins its maturation cycle during the 6th week of pregnancy, followed by the superior visual colliculus that begins maturation around the 8th week. However, the development cycle is fairly protracted, as the inferior colliculus does not fully form until about the 17th week, and the superior colliculus takes several weeks longer to establish. Furthermore, midbrain development and myelination are not nearly complete until well after birth. Around GW36, the inferior-auditory colliculus, in collaboration with the lower brainstem, is capable of auditory discriminations and responds to sounds by accelerating the fetal heart rate (FHR), as well as with eye and head movements. The fetus hereafter reacts with reflexive movements when stimulated and is also able to fall asleep with the appearance of rapid eye movements (REM). Therefore, auditory discrimination, FHRs, the wake-sleep cycle, positioning, and protective behaviors appear to be controlled reflexively by the brainstem, which also appears to be engaged in learning processes. These processes have been thoroughly described elsewhere (Huang et al., 2009; Dubois et al., 2014; Seto and Eiraku, 2019).

### Neurogenesis and neuronal migration

**FIGURE 3:**
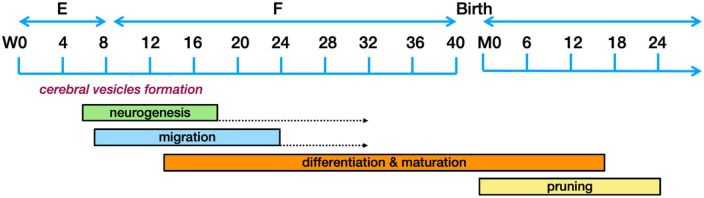
Schematic representation of the human brain development timeline. The dotted arrows designate a reduction in the rate of neurogenesis. Figure adapted from Stagni et al. (2015), obtained by Elsevier (No. 5410821023270) under a creative commons license 3&4.

The different components of the nervous system are developed in distinctive phases going from (1) neurogenesis to (2) neuronal migration to the adequate location, (3) their differentiation along with connection maturation, and (4) the pruning of connections. These developmental phases are governed by hormones, specific proteins and growth factors, which function as traffic cues for the cells or their related processes. These intricate processes have been extensively reviewed (Huang et al., 2009; Lui et al., 2011; Dubois et al., 2014; Kostoviæ et al., 2015; Adnani et al., 2018; Hadders-Algra, 2018a; Lennox et al., 2018; Cadwell et al., 2019; Seto and Eiraku, 2019) and only those aspects that could possibly have relevance to ASD pathogenesis will be explored here. For clarity, Figure 3 presents a timeline of human brain development.

As stressed above, the cerebral cortex of the adult mammal has a stratified structure made up of different layers, each of which containing a combination of glutamatergic pyramidal neurons, interneurons and projection neurons, and non-pyramidal GABAergic neurons. The great majority of cortical GABAergic cells are extraordinarily varied interneurons that form local connections only, whereas pyramidal neurons account for around 80% of all adult cortical neurons (Wang Y. et al., 2018).

The pallium and subpallium neuroepithelial stem cells (NESCs) multiply extensively by GW5–6 in humans to generate the cerebral vesicles (Bystron et al., 2008). NESCs maturate into radial glia cells, the key progenitor cells in the developing nervous system. Extracerebral microglial cells invade the telencephalon from GW5 onward and regulate the neurogenetic process (Nie et al., 2010). During GW5–7, the first neurons to be generated from the heterologous and transient cell populations of both pallial and subpallial origins are the CR cells and the subplate cells (SP) (Barber and Pierani, 2016). CR cells are a transient cell population that is critical for brain development: these neurons form the preplate (PP) during GW6–7, and are devoid of synapses, communicating through non-synaptic junctions (Kostoviæ et al., 2015). Positioning cues and instructions to the developing cortical neurons and afferents are provided by reelin secreting CR and SP cells (Lakatosova and Ostatnikova, 2012; Barber and Pierani, 2016). The latter regulate migration of developing neurons that will form the thalamocortical connections (Ghosh et al., 1990; Csillik et al., 2002). Through a NMDA receptor-controlled process, reelin secreted by CR cells guides the direction of migration and positioning of neurons (Schaefer et al., 2008; Wang S. et al., 2018). CR cells also play an essential role in corticogenesis by regulating the identity and function of radial glia and the radial glia-to-astrocyte transformation (Super et al., 2000).

**FIGURE 4:**
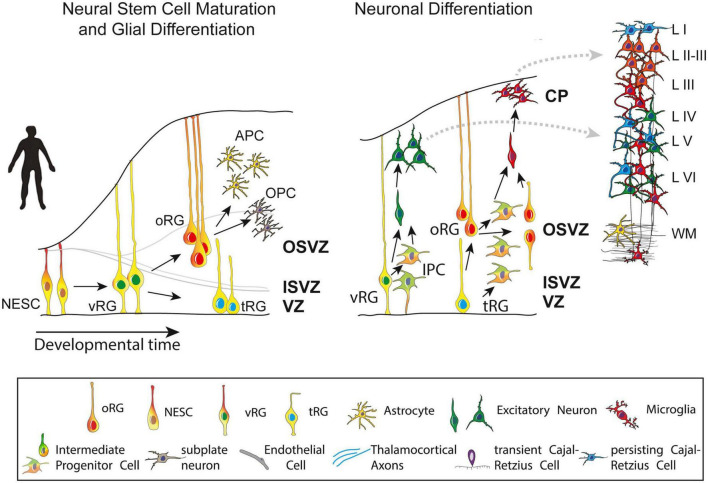
Maturation and differentiation in the cortex. **(Left)** Schematic representing radial glia maturation from neuroepithelial stem cells (NESCs), followed by their differentiation into astrocytes. **(Right)** Schematic represents sequential production of cortical layers from radial glia. Human cortical development involves an expanded diversity of radial glia with distinct maturation trajectories **(left)**. Neurogenesis in the human cortex occurs in the ventricular zone early in development and progressively shifts toward the outer subventricular zone (OSVZ). The development of the OSVZ is not homogeneous across prospective cortical areas in human fetuses, but increases from prospective mesocortical to prospective isocortical areas (Barbas and García-Cabezas, 2016). IPCs, intermediate progenitor cells; ISVZ, inner subventricular zone; LI–VI, cortical layer I–VI; NESCs, neuroepithelial stem cells; oRG, outer radial glia; OSVZ, outer subventricular zone; SVZ, subventricular zone; tRG, truncated radial glia; vRG, ventricular radial glia; VZ, ventricular zone; WM, white matter. Figure adapted from Cadwell et al. (2019).

Post-mitotic excitatory pyramidal neurons migrate along radial glial (RG) fibers in an inside-out gradient of development (the deepest cellular layers are built first, followed by the ones closest to the surface) from layer VIa to layer II to produce the cortical plate by GW7–20 (Figure 4). Pyramidal cells originate from radial glial cells (RGC) at the beginning of cortical plate development (GW7–8), but later, they mostly come from intermediate progenitor cells (IPC) or basal progenitors derived from RGC cells (Kriegstein and Noctor, 2004; Corbin et al., 2008). The first pyramidal neurons emerge successively from the ventricular zone (VZ) of the cortex, from which they migrate radially to shape a layer within the PP, i.e., the cortical plate (CP), dividing the PP into two zones: the superficial marginal zone (MZ; presumptive layer I containing the CR cells) and the deep SP.

The subventricular zone (SVZ) is a proliferative zone that forms during GW7, while the cortical plate emerges during GW8, consisting of a cell-dense area with post-migratory neurons that migrate from the VZ along radial glial guides and eventually remain in the CP, arranged in vertical ontogenetic columns. *These columns are characteristically disorganized in the ASD cortex*.

The neocortical anlage is thus constituted of three transient fetal zones: the cell-poor superficial MZ, the cell-dense CP and the plexiform pre-subplate. Early bilaminar synaptogenesis is also found in this neocortical anlage, with synapses present in the MZ and pre-subplate, but absent in the CP. The intermediate zone (IZ) occurs between the neocortical anlage and the periventricular proliferative zones (VZ-SVZ) and comprises early developing afferent fibers emanating from the brain stem (monoaminergic axons), the thalamus (glutamatergic axons) and basal forebrain (cholinergic axons) (Kostoviæ et al., 2015; Figure 4). For further details the reader may refer to (Smart et al., 2002; Huang et al., 2009; Lui et al., 2011; Reillo et al., 2011; Martínez-Cerdeño et al., 2012; Dubois et al., 2014; Kostoviæ et al., 2015; Adnani et al., 2018; Hadders-Algra, 2018a; Lennox et al., 2018; Cadwell et al., 2019; Seto and Eiraku, 2019).

### Neuronal organization and functionality

The brain undergoes the most substantial changes from the second half of pregnancy to the first three months after birth, particularly in the cortical subplate and cerebellum. From early fetal life to the first trimester post-partum, the transient SP undergoes constant developmental changes and interactions, with clear functional activity. From then on, a second developmental phase becomes distinct, where permanent circuitries dominate the now formed cortical SP (Kostoviæ et al., 2015). The first phase appears to correspond to non-adaptive, exploratory motor behavior and the second to motor behavior integrating environmental restrains (Hadders-Algra, 2018b). *Disruption of SP development is considered to have a great impact in developmental disorders such as schizophrenia, cerebral palsy, ASD and attention deficit hyperactivity disorder (ADHD)* (Hadders-Algra, 2018a).

**FIGURE 5:**
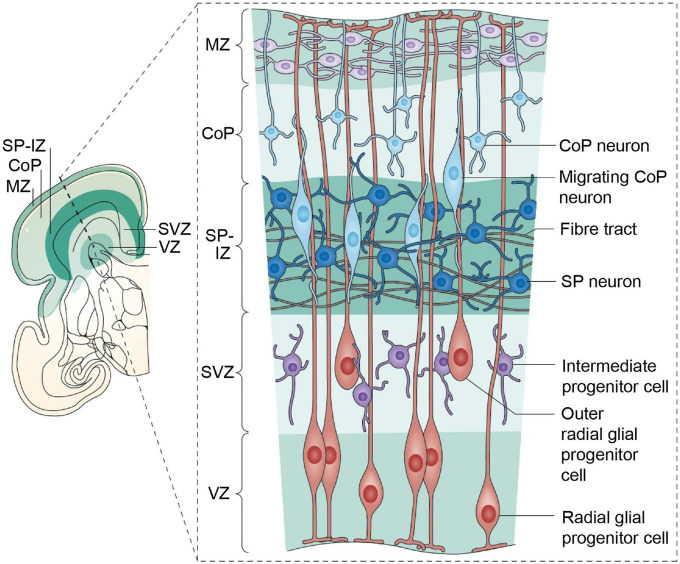
The human cerebral cortex at 28 weeks postmenstrual age (PMA; 8 weeks before birth). A coronal section is shown on the left; the inset box on the right provides details of the developmental processes. The ventricular zone (VZ) and subventricular zone (SVZ) constitute the germinal matrices where cell division occurs. The first generations of cells are generated in the VZ, the later generations in the SVZ. The SVZ is a structure that expanded during phylogeny; it is especially large in primates. The radial glial cells span their shafts between the germinal layers and the outer layer of the cortex [marginal zone (MZ)]. The first-generation neurons have migrated to the subplate (SP) and participate in the functional fetal cortex; later generations of neurons migrate to the cortical plate (CoP). Figure from Hadders-Algra (2018a), see Luhmann et al. (2018) for a detailed review, obtained by Elsevier (No. 5410821023270) under a creative commons license 3&4.

During the first phase of SP development, SP neurons (SPNs; Figure 5) are among the first populations to be generated in the cortex (Kostovic and Rakic, 1980) and to receive synaptic inputs from thalamic axons. These function as a temporary link between thalamic axons and their final target in layer IV (Friauf et al., 1990; Ghosh and Shatz, 1992), that will be invaded and innervated only later in development (Ghosh and Shatz, 1992; Kanold et al., 2003). It is important to note here that the monoamine serotonin (5-HT), a crucial regulator for the formation of neural circuits (*see below*), presynaptically suppresses thalamo-SP afferents via 5HT1B receptors, although without apparently influencing intrinsic SPN properties (Liao and Lee, 2012).

The subplate neurons (SPNs) have a critical function in founding the correct wiring (Casal and Romano, 1978; McConnell et al., 1989) and functional maturation (Kanold et al., 2003) of the cerebral cortex; they then disappear during postnatal development. SPNs appear to be selectively sensitive to injuries (such as hypoxia) that, in humans, are linked to motor and cognitive defects (McQuillen and Ferriero, 2005).

### The effects of serotonin on fetal brain development

**FIGURE 6:**
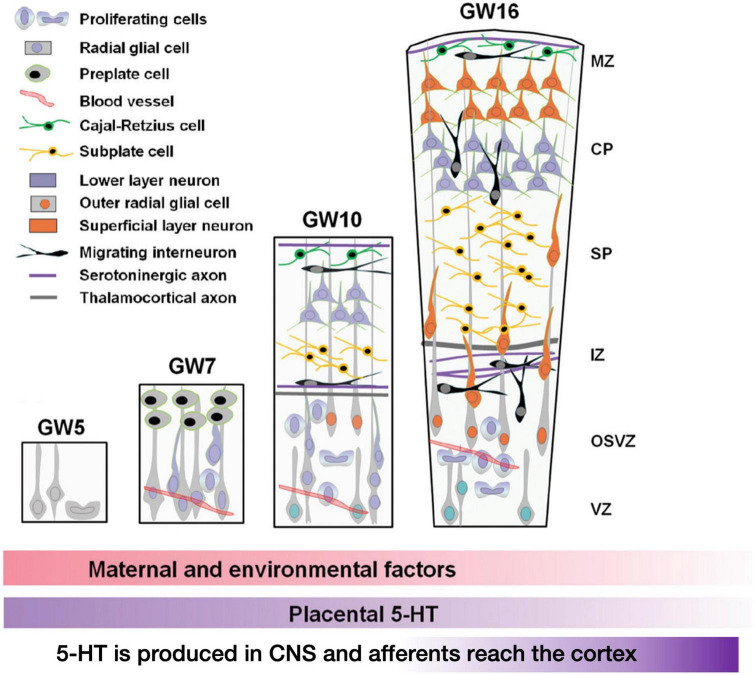
Early stages of development of the human cerebral cortex in relation with 5-HT afferents. In human, intense proliferation of the neuroepithelium and formation of the preplate (PP) take place around GW5 and GW6–7, respectively. By GW8–10, PP is split by the migration of the first pyramidal neurons. Cajal-Retzius cells (C-R) will remain in the marginal zone (MZ, presumptive layer I) while sub-plate neurons (SP) will be positioned below the cortical plate (CP). In addition, around GW10, another source of progenitors arises: the outer radial glial (oRG) cells that do not maintain contacts with the apical surface. Monoaminergic axons and thalamo-cortical axons (TC) are already found in the MZ and in the intermediate zone (IZ), respectively. By GW16, SP cells occupy a large proportion of the cortical anlage and oRG are still producing a high number of neurons. Interneurons migrating first tangentially and later radially to the pial surface, incorporate into the CP. The cortical structure is vascularized very early and carries platelets and mast cells that could provide 5-HT to the developing embryo. During the initial phase of cortical development, 5-HT is mainly synthesized in the placenta while later produced by serotonergic neurons of the embryo. The colored lower bars indicate the times at which different factors (maternal and environmental; 5-HT of placental origin, 5-HT produced by the embryo itself) could affect the development (figure from Vitalis and Verney, 2017), obtained by Elsevier (No. 5410821023270) under a creative commons license 3&4.

Higher-order cognitive functions are controlled by cortical circuits, and their operation is heavily reliant on their structure, which evolves during development. The proliferation, migration, and differentiation of neurons and glial cells are among the sequential events that occur primarily during embryonic and early post-natal development and culminate in the establishment of mammalian cortical circuits. While these systems are mostly controlled by genetic programming, they are also affected by a wide range of extrinsic signals (such as hormones, growth factors, guidance cues, cell adhesion molecules, and so on) as well as environmental influences. As hinted before, serotonin (5-HT) is a critical regulator for the development of neuronal circuits (Gaspar et al., 2003; Bonnin and Levitt, 2011). 5-HT influences embryonic development very early, even before serotoninergic neurons appear (Lauder and Krebs, 1978; Shuey et al., 1992; Yavarone et al., 1993; Moiseiwitsch and Lauder, 1995; Whitaker-Azmitia et al., 1996; Buznikov et al., 2001). In the developing embryonic cortex, a number of serotonin receptors are dynamically expressed in a cell-type-and region-specific way. The ionotropic receptor 5-HT3A and the metabotropic receptor 5-HT6 control diverse elements of cortical architecture, such as neuronal migration and dendritic differentiation. Most of the 5-HT reaching the embryo at these early developmental stages originates from maternal or placental sources (Figure 6 below recapitulates these studies), while increased placental L-tryptophan conversion to 5-HT as a result of maternal inflammation during pregnancy disrupts 5-HT-dependent neurogenic processes during fetal neurodevelopment (Goeden et al., 2016; Williams et al., 2017; Shallie and Naicker, 2019).

### Serotonin and neuronal migration

In the mammalian cortex, 5-HT not only regulates the migration of cortical neurons but also triggers the motility of microglia toward the Central Nervous System (CNS) (Krabbe et al., 2012). Secreted or exogenous 5-HT acts at the molecular level as an axon guidance cue to shape neuronal circuit formation during development (Vicenzi et al., 2021). 5-HT produces concentration-opposite effects: during the late phase of neuronal migration, interneurons expressing 5-HT3A receptors that derive from the caudal ganglionic eminence (CGE) respond increasingly to *low* receptor activation in order to migrate radially toward and integrate into the cortical plate. This type of response increases 5-HT3A positive interneuron motility and enhances radial migration and growth cone activity while defining the positioning of their subpopulations (Murthy et al., 2014). Importantly, it further leads to long-lasting changes in the positioning of reelin positive cortical interneurons (Murthy et al., 2014). Likewise, *ex vivo* application of a high 5-HT concentration on cortical slices decreased the migration rate of both GABA and non-GABAergic neurons (Riccio et al., 2011). High 5-HT further resulted in the retraction of GABAergic neuron migration toward the intermediate zone and cortical plate. This effect was largely mediated by the activation of the cAMP signaling pathway through the 5-HT6 receptor (Riccio et al., 2009) while the same behavior was also reported for glutamatergic neurons (Dayer et al., 2015). In general, increased 5-HT levels modify activity-dependent segregation mechanisms during brain development: KO mice lacking the gene encoding the serotonin transporter (SERT) or the monoamine oxidase A (MAOA) exhibit increased 5-HT levels and the segregation of ipsilateral and contralateral regions in dorsal lateral geniculate nucleus (dLGN) does not occur properly (Upton et al., 1999, 2002; Gaspar et al., 2003; García-Frigola and Herrera, 2010).

Serotonin additionally affects the function of CR cells, which are central regulators of cortical development, and especially of dendritic arborization. Arborization is controlled by the secretion of the glycoprotein reelin (Super et al., 2000; Lakatosova and Ostatnikova, 2012), secretion of which is partly regulated by the quantity of 5-HT present in the area (Vitalis et al., 2013). During embryonic development, CR cells receive serotonergic and noradrenergic synaptic inputs (Janusonis et al., 2004), while the catecholaminergic system projects to the forebrain about the same time as the 5-HT system (E11.5 in mice and approximately GW9 in humans) (Kalsbeek et al., 1988, 1990). Around E15 (approximately GW14 in humans), two streams of tyrosine hydroxylase (TH)-positive axons (i.e., TH is a marker for dopamine, norepinephrine, and catecholamine positive neurons, as it is the rate limiting enzyme for dopamine synthesis) from the rostral section of the ventral tegmental area (VTA) enter the PFC, one in the subplate (SP) and the other in the marginal zone (MZ), where the CR cells reside (Bodea and Blaess, 2015).

Within the medial PFC (mPFC), the TH and 5-HT fibers are found in proximity to reelin-secreting CR cells. Altered 5-HT signaling modifies prefrontal cell characteristics, catecholaminergic innervation and cortical integrity (Garcia et al., 2019) suggesting that, during development, there is a functional interplay between 5-HT and catecholaminergic systems that modulates the cellular architecture of the PFC. These two systems have significant structural overlap in the adult brain and appear to receive inputs from each other (Pollak Dorocic et al., 2014). Furthermore, given their active role in higher-order cognitive functioning, there is a clear interplay between the 5-HT and catecholaminergic projections to the PFC (Bortolozzi et al., 2005; Di Giovanni et al., 2008; Niederkofler et al., 2015).

In the mouse, an excess of 5-HT at embryonic day E17.5 (approximately GW17–18 in humans), the period where cortical interneurons and pyramidal neurons migrate to form upper cortical layers, is found to alter their migration in a reversible way (Vitalis et al., 2013). Furthermore, pharmacological perturbation of the 5-HT system by 5-methoxytryptamine, that is a non-selective 5-HT receptor agonist, lowers the levels of reelin that circulate in the blood flow at birth (Janusonis et al., 2004) and results in abnormal microcolumns formation in the presubicular cortex of mice by postnatal day 7. This is a characteristic commonly seen in the brains of ASD patients, along with lower levels of reelin, suggestive of CR cells involvement in the disorder (Fatemi et al., 2005; Lakatosova and Ostatnikova, 2012; Folsom and Fatemi, 2013). Early-life serotonin dysregulation appears to irreversibly impact cortical microcircuit formation and to largely contribute to the occurrence of psychiatric phenotypes.

**FIGURE 7:**
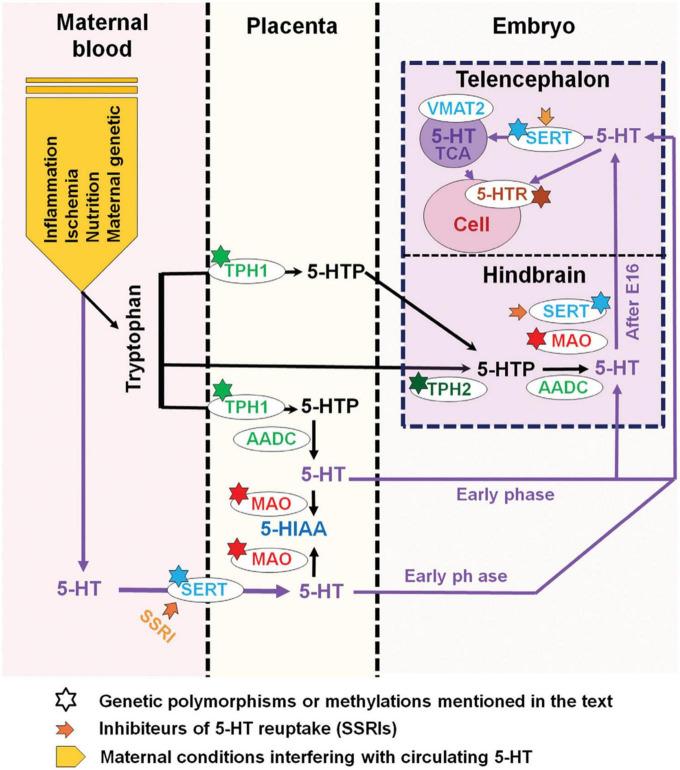
Maternal, placental, genetic, and pharmacological conditions determining the amount of serotonin supply to the developing telencephalon. The placenta provides tryptophan to the embryo but can also convert it into 5-HTP (5-hydroxytryptamine) or further into serotonin (5-HT) via various maternal metabolic enzymes. In addition, 5-HT from maternal sources could be taken up by the placenta where the serotonin transporter (SERT) is also expressed. During early embryonic stages, 5-HT could thus be directly delivered to the developing embryo. In the mouse, after E15-E16 (approximately GW 16 in humans), when 5-HT axons of the hindbrain reach the cortex, 5-HT could act on various target cells expressing selected arrays of 5-HT receptors. At this stage, 5-HT could also be taken up and stored by thalamocortical afferents (TC) and released after specific stimulation. In addition, 5-HTP is provided by Tph2 (tryptophan hydroxylase type 2) and AADC (aromatic amino acid decarboxylase) containing neurons that synthetize 5-HT. The large downward arrow on the left in this diagram, adapted from Bonnin and Levitt (2011), points to the maternal conditions that are best known to interfere with 5-HT availability to the embryo. Inhibitors of 5-HT uptake (SSRIs) which affect SERT function at all levels are indicated by a thick orange arrow while known genetic polymorphisms or epigenetic alterations (methylations) which impact serotonin supply are indicated by a star. The major catabolic enzymes of 5-HT are MAO (monoamine oxidases) and tryptophan hydroxylase type 1 (Tph1). Adapted from Bonnin and Levitt (2011) and Vitalis and Verney (2017), obtained by Elsevier (No. 5410821023270) under a creative commons license 3&4.

Serotonin supply to the fetus during the first half of pregnancy mostly relies on exogenous sources, i.e., the placental and maternal platelets that transfer 5-HT from the gut. Serotonin-dependent neurodevelopmental activities, such as corticogenesis and circuitry maturation, coincide with this time frame. As a result, prenatal variables that affect both externally derived and raphe-produced 5-HT content can have a significant impact on the offspring’s early brain development (Hanswijk et al., 2020; Figure 7). It is interesting to note that the most consistent neurochemical finding reported in ASD affected individuals is a platelet hyperserotonemia, with a 40–70% increase in platelet 5-HT occurring in as many as 30% of autistic subjects (Veenstra-VanderWeele et al., 2002; Janusonis, 2005; Whitaker-Azmitia, 2005; Azmitia et al., 2011).

### Maternal inflammation and autism spectrum disorder

Maternal inflammation during pregnancy has an effect on placental function and is linked to an increased risk of neurodevelopmental problems in children. There are indications from epidemiological studies that, during pregnancy, maternal infections by a wide variety of agents, including viruses such as influenza (Shi et al., 2003; Brown et al., 2004) and rubella (Shi et al., 2003; Brown et al., 2004), bacteria (bronchopneumonia; Sørensen et al., 2009), and protozoa (*Toxoplasma gondii*; Brown et al., 2005) increase the risk of schizophrenia or ASD (Grønborg et al., 2013; Zerbo et al., 2015; Meltzer and Van de Water, 2017) in the offspring. This might be reflected in ASD seasonality, associated with a 6% increased risk for children conceived during the winter months (Zerbo et al., 2011).

Moderate immune activation during gestation upregulates tryptophane (TRP) in circulation and its subsequent placental conversion to 5-HT by TRP hydroxylase (TPH). This increases exogenous 5-HT delivery to the fetus and leads to the restriction of 5-HT axonal outgrowth during fetal forebrain development (Goeden et al., 2016). Temperate systemic immune challenge in the mother by the immunostimulant polyriboinosinic-polyribocytidylic acid [Poly (I:C)] (Goeden et al., 2016) rapidly upregulates placental TRP conversion to 5-HT in the mouse, leading to accumulation of exogenous 5-HT within the fetal forebrain, blunting endogenous 5-HT axonal outgrowth specifically (Goeden et al., 2016; Kliman et al., 2018).

Conversely, during intrauterine inflammation, the upregulation of placental indoleamine 2,3-dioxygenase (IDO) shunts tryptophan metabolism away from 5-HT and toward the kynurenine pathway, with neuroactive metabolites being released in fetal circulation (Dharane Neé Ligam et al., 2010). In the fetal brain, kynurenine is converted to kynurenic acid (KA) (Notarangelo et al., 2019), an endogenous antagonist of both NMDA receptors (NMDARs), expressed by SP neurons, and α7-nicotinic receptors expressed by CR cells (Csillik et al., 2002). NMDARs regulate tangential migration in the developing cortex (Ghosh et al., 1990; Csillik et al., 2002) while α7-nicotinic receptors guide the direction of neuronal migration and positioning (Schaefer et al., 2008; Wang S. et al., 2018). In the rabbit, intrauterine inflammation leads to significant upregulation of IDO in the placenta with intense microglial activation and increases in kynurenine acid and quinolinic acid, followed by a significant drop in 5-hydroxyindole acetic acid (a precursor of serotonin) levels in fetal brain’s periventricular region at G29 (24 h after treatment) (Williams et al., 2017).

Low grade systemic inflammation is reported to be as high as 25% among US women (Ford et al., 2004), while the gestational period is further subject to maternal inflammation due to numerous phenomena involving fetal genotypes and environmental factors that affect maternal well-being. Maternal infection and/or increased 5-HT supply to the developing fetus during pregnancy, while clearly promoting ASD pathogenesis, is only associated with higher risk of autism and not with overt ASD pathogenesis (Zerbo et al., 2015; Brucato et al., 2017; Guisso et al., 2018). However, it is also clear that increased 5-HT supply to the developing fetus cannot, by itself, be the main inflammatory-associated factor that could be implicated in ASD pathogenesis. Indeed, many other inflammatory mediators, such as the interferons, various interleukins, TGF-β, TNF-α, and β, etc., can reach fetal circulation from the placenta and have been shown to significantly affect brain development during gestation (Wei et al., 2013; Theoharides et al., 2016; Tsivion-Visbord et al., 2020). Furthermore, various studies suggest that the neurodevelopmental consequences of maternal inflammation are depended on the type of the ongoing infection, the gestational time at which the inflammatory reaction starts, its intensity and its duration (for example, see Boksa, 2010). The placenta may react to maternal inflammation with sexually dimorphic consequences for fetal development (Clifton, 2005; Mueller and Bale, 2008) while testosterone surges in newborn males can alter long-term CNS function (Forgie and Stewart, 1993; Wilson and Davies, 2007).

### Columnar structure of the neocortex: Typical development versus autism spectrum disorder

**FIGURE 8:**
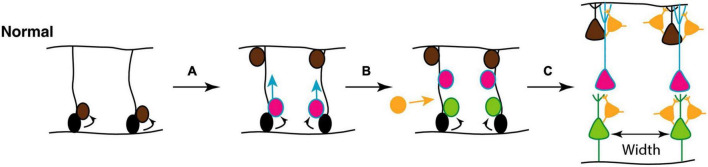
**(A)** Mechanisms underlying focal organization and minicolumn distribution in the cortex. During normal cortical development, radial glial neural stem and progenitor cells (NSCs; black) located in the ventricular zone, with radial processes extending to the pial surface of the neural epithelium, can undergo symmetrical divisions to produce more radial glial NSCs (brown) or asymmetric divisions **(A)** to maintain the NSC pool and generate committed neural progenitors (pink); **(B)** the latter migrate toward the pial surface on the radial scaffold, where they differentiate into projection neurons. This process continues with different classes of neurons (pink, green) being produced in successive waves over developmental time. Hence, different neuronal types are grouped together in different cortical layers **(C)**. GABAergic interneurons (orange) migrate into the neuroepithelium **(B)**, and integrate into the circuitry **(C)**. At the end of this process, projection neurons are organized in radial minicolumns. Figure adapted from Donovan and Basson (2017), obtained by Elsevier (No. 5410821023270) under a creative commons license 3&4.

As described above, during neurogenesis, newly generated projection neurons migrate along radial glial fibers from the ventricular layers toward the outer, pial surface, giving rise to a modular organization in which cell bodies in the neocortex are ‘stacked’ on top of one other to construct so-called minicolumns. They consist of vertical chains of approximately 80–100 neurons between cortical layers II–VI, with associated dendrites and bundles of myelinated axons. They serve as the basis for cortical information processing by allowing thalamic inputs to be combined with corticocortical processing and thus provide modulatory inputs to incorporate behavioral context into sensory processing (Opris and Casanova, 2014; McKavanagh et al., 2015). The final spacing of these minicolumns is determined by the density of glial scaffolds during development (Figure 8).

**FIGURE 9:**
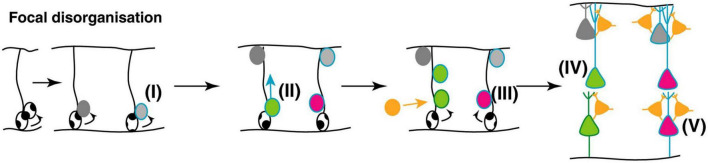
Potential mechanisms underlying focal disorganization and altered minicolumn distribution in ASD cortex. Focal disruptions may be the result of a combination of early genetic, epigenetic changes or environmental changes affecting neural stem and progenitor cell such that the resulting neuronal progenitors have defects in **(I)** differentiation **(II)** or migration **(III)** causing them to adopt the wrong fate **(IV)**, or end up in an in appropriate position in the brain **(V)**. The end result is a cortex with apparent disorganization of neuronal cell types and distorted cell densities. Figure adapted from Donovan and Basson (2017), obtained by Elsevier (No. 5410821023270) under a creative commons license 3&4.

In ASD affected individuals, there is apparent focal disorganization, with the minicolumns being spaced farther apart with a lower cell density in a given region of cortex (Donovan and Basson, 2017) (Figure 9). Although the effect appears to affect higher order association areas most significantly, a 6–10% difference in minicolumn width is observed across all cortical regions (Casanova et al., 2006; McKavanagh et al., 2015; Donovan and Basson, 2017).

This phenomenon suggests anatomical misplacement of the reelin secreting CR cells which provide the positioning cues to the developing cortical neurons and afferents. Hence, while the normotopic position of reelin is important for proper neuronal migration and positioning, leading to their characteristic lamination, it does not affect radial glial elongation (Schaefer et al., 2008; Wang S. et al., 2018). As exposed above, 5-HT regulates the physiology of CR cells through transient synaptic contacts with serotoninergic projections, while reelin secretion is regulated in part by the amount of brain 5-HT (Janusonis et al., 2004). Hence, one of the neuroanatomical phenomena that characterize ASD cortical structures, namely disorganized and altered cortical minicolumn distribution, appears to find its roots very early during fetal brain development, possibly as a result of excess 5-HT and/or kynurenine supply to the developing fetus. This may arise subsequently to a sustained low-level maternal inflammatory reaction during a critical developmental window starting around GW9-10 and probably extending to GW20 (see Figure 9).

### Dendritic and synaptic development

**FIGURE 10:**
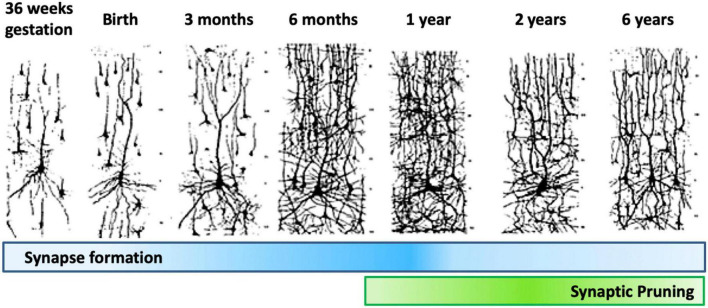
Synapse formation and pruning in neurotypical development. Starting from birth until adolescence, the synaptic pruning process, preceded by synaptic overgrowth, optimizes the neurological network by removing redundant and weak connections as defined by the learning process, effectively making the brain more efficient. Adults have been found to have approximately 41% fewer neurons than newborns (Abitz et al., 2007). Figure adapted from Conel (1939), obtained by Elsevier (No. 5410821023270) under a creative commons license 3&4.

Dendrite development progresses through dendritic arborization and spine growth. The dendrites protrude from the cell body as individual processes. Their development begins prenatally in humans, and continues for about 2 years postnatally, with different phases of synaptic density being detected in the cerebral cortex of primates. The first phases, which occur during early embryonic development, generate low density synapses. Then, synapse number progresses rapidly until nearly the 2nd year of age. Moving forward, the number of synapses reaches a plateau, followed by a rapid synapses elimination phase which continues through adolescence. The last phase is characterized by a new plateau in synapse number in adulthood, followed by a drop through aging. The initial synapse reduction period is acute, dropping to 50% of the number present at 2 years of age through synaptic pruning (Figure 10).

### Serotonin and post-natal synaptic pruning: Synaptic pruning defects in autism spectrum disorder

Proper synaptic pruning during postnatal development is essential for the development of functional neuronal circuits. When the synaptic pruning process is impaired, the excitatory versus inhibitory balance (E/I balance) of synapses is disrupted, and this may cause neurodevelopmental disorders such as ASD. The process of synaptic pruning is mostly handled by microglial cells that are -in general- adept to promote neuronal death and clear apoptotic cells (Mallat et al., 2005). During brain development, they are the key players in the elimination of redundant and weak synapses, thus ensuring the proper wiring of neuronal networks. They engulf inappropriate and less active synapses through microglial autophagy, achieving synaptic refinement and neuro-behavioral regulation through selective reshaping of axonal and dendritic arborizations (Paolicelli et al., 2011; Paolicelli and Gross, 2011; Koyama and Ikegaya, 2015). The process appears to involve the neuronal chemokine fractalkine and the CX3CR1 microglial receptor for microglia recruitment and activation (Sheridan and Murphy, 2013). Serotonin is also involved both directly and indirectly in this developmental process (Okado et al., 2001; Serfaty et al., 2008; Penedo et al., 2009; Lesch and Waider, 2012). Activation of 5-HT receptors promotes microglial injury-induced motility but attenuates phagocytic activity (Krabbe et al., 2012). Indeed, postnatal microglia from mice lacking the 5-HT2B receptor (Htr2B^–/–^) are unable to reach serotonergic synaptic boutons, unlike microglia from wild-type mice (Krabbe et al., 2012). This was confirmed prepartum in the *in vivo* developmental model of dLGN synaptic refinement where Htr2B^–/–^ mice had anatomical alterations of retinal projections into the thalamus (Kolodziejczak et al., 2015).

**FIGURE 11:**
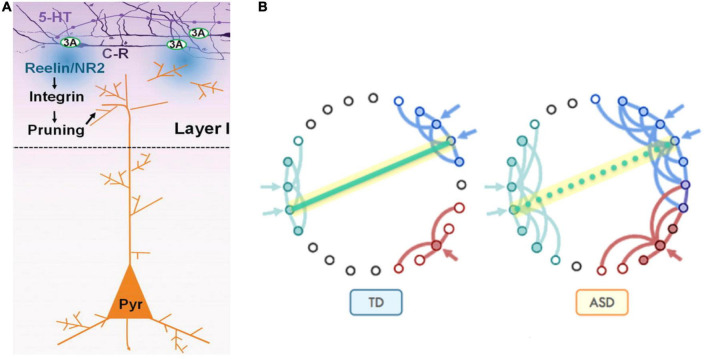
**(A)** Synaptic pruning in brain development serves to refine communication between functional areas by improving the signal-to-noise ratio. At early postnatal stage, Cajal-Retzius cells (C-R) that express 5-HT3A, respond to 5-HT by releasing reelin which, through the activation of the integrin signaling pathway, induces pruning of apical dendrites of pyramidal neurons (Pyr). **(B)** Pruning reduces unnecessary cross-talk with neighboring nodes (blue and red in typical development, Panel TD). However, in the presence of too many overlapping connections, distinct signals from neighboring communities may compete and interfere with each other, effectively creating noise and “confusion” within the network (blue and red in autism spectrum disorder, Panel ASD), while negatively affecting the development of long-distance connectivity, such as those between fronto-parietal regions for higher-order executive functions [adapted from Belmonte et al. (2004), Just et al. (2012), and Vitalis and Verney (2017)], obtained by Elsevier (No. 5410821023270) under a creative commons license 3&4.

Serotonin also indirectly influences the synaptic pruning process. 5-HT overstimulation during *in utero* development result in misplacement of apical dendrites with respect to the post-natal source of reelin (CR cells) and abnormal cortical microcolumns formation. This culminates in both apical dendrites’ exuberance (preservation due to distancing from the source of reelin) and excessive pruning as a consequence of close contact with the source of reelin, depending on the local forms of earlier neuronal migration patterns dysregulations (Figure 11).

During the first two decades of life, where synaptic pruning is active, cortical thickness (CT) is likely to represent dendritic arborization. As a result, CT maturational alterations are mostly related to dendritic pruning. However, in autistic individuals, there is a dramatic increase in dendritic growth during the first year of life, while the synaptic pruning process happens to a far lower degree, with a large number of “redundant” connections remaining intact. By 2 years of age, the minicolumns are spaced farther apart with a lower cell density in the cortex. The space between minicolumns is occupied by dendritic bundles and axonal fascicles that extend into multiple layers of the cortex. By late childhood spine density of neurons drops by 50% in TD brain, whereas by only about 16% in ASD brains, negatively impacting signal-to-noise discrimination (Tang et al., 2014; Lieberman et al., 2019). This is reflected by the slower rate of cortical thinning between infancy and adolescence in ASD compared to TD, with an increased thinning in late adolescence, whereas in TD, thinning occurs around puberty and early adulthood (Nunes et al., 2020).

Abnormal pruning is further corroborated by the reduced level of reelin (Reln) and Dab 1 (reelin signal transducer) proteins and mRNA levels in frontal and cerebellar, and non-significantly in parietal, areas of autistic brains compared to control subjects. Accordingly, the mRNA for the reelin receptor (VLDLR) is found significantly elevated in superior frontal and cerebellar areas of autistic brains compared to controls (Fatemi et al., 2005; Kelemenova and Ostatnikova, 2009).

Because the cortex is heavily implicated in autistic behaviors, a lack of synaptic pruning in this area will have an impact on the severity of autistic behaviors, as well as on the loss of abilities. Therefore, dysregulation of post-natal synaptic pruning as a result of neuronal migration patterns alterations during the key stages of cerebral cortex early development (between week postnatal WP8 and WP20) stands to result in a huge variety of forms and degrees of signal-over-noise (S/N) discrimination losses, thereby accounting for the enormous clinical heterogeneities encountered in a variety of neurodevelopmental cognitive disorders, prominent amongst which is ASD.

### RNA editing mechanisms and epigenetic traits

It is important to stress that while the neuro-anatomical characteristics of ASD may, possibly, be induced by changes in exogenous 5-HT/kynurenine supply, individual susceptibility could be best explained either through epigenetic marking alterations, or dysregulated RNA editing mechanisms. While epigenetic markings may directly affect the relative expression levels of individual genes or groups of genes, they however, require environmental conditions to stay stable long enough to have real physiological effects. Therefore, their relative weight compared to RNA editing mechanisms during brain development is expected to be secondary. RNA editing, in association with both genotypes and environmental conditions, could alter spatiotemporal proteins expression patterns at both quantitative and qualitative levels, in a tissue-specific and context-dependent manner.

As 5-HT signaling is dependent on numerous receptors, transporters, enzymes, other neurotransmitters, and associated genes, a single gene or a cluster of genes cannot account for the consequences related to 5-HT fluctuations. The 5-HT system contains multiple classes of receptors (5-HT1–5-HT7) and related subtypes, some of which present allelic variations that influence receptor function and availability (e.g., 5-HT1A; Parsey et al., 2006), while others are subject to combinatorial mRNA editing, which affects not only receptor and ion channels function and availability, but also expression patterns (e.g., 5-HT2C; Eran et al., 2013). Extensive RNA editing, comprising site-dependent, selective splicing alterations, polyA-tails deadenylation and adenosine-to-inosine (A-to-I) RNA editing (Richter, 2007; Raj and Blencowe, 2015; Behm et al., 2017), takes place in the brain, and a developmentally increasing editing pattern from fetal to adult samples has been reported. This increase in A-to-I editing pattern was associated temporally with the growth of cortical layers and with neuronal maturation (Hwang et al., 2016). Furthermore, allelic variants encoding proteins such as SERT (SLC6A4; transports serotonin from synaptic spaces into presynaptic neurons and terminates serotonin activity; the S-allele is particularly important for ASD; Brummelte et al., 2017) and CPEB4 (which governs mRNA polyA-tails dynamics) as well as allelic variants of ADAR1 and 2 (which govern A-to-I RNA editing) can significantly affect brain development, both in a positive as well as a negative manner^1^. Indeed, CPEB4 binds to the transcripts of most high-confidence ASD-risk genes (Parras et al., 2018), while A-to-I RNA editing addresses neuronal receptors and channels organization (Streit and Decher, 2011). The latter mostly affects glutamate and voltage-gated ion channels, GABAA and serotonin 2C receptors, filamins and actin organization, while inducing changes in miRNA profiling that affect dendritic spine density, neuronal organization and synaptic transmission by altering the interaction profiles with binding partners (Scadden and Smith, 2001). The detailed results of our work on the impact of RNA editing mechanisms in the pathogenesis of ASD will be the subject of a specific publication.

### The likely consequences of defective post-natal synaptic pruning: Focus on the autonomic nervous system

Defects in synaptic pruning may well underlie the variability in S/N, represented by the long-distance underconnectivity and local overconnectivity observed in ASD patients (Courchesne and Pierce, 2005; Anderson et al., 2011; Keown et al., 2013), while also accounting for the large heterogeneity between individuals and age groups.

The exuberance of thin axons that travel short to medium distances in autism, as observed in areas underlying the prefrontal limbic areas, alters the S/N threshold and could ‘scramble’ long-distance communication signals. This connectivity bias could provide the anatomical basis for the disrupted transmission of emotion signals and help explain why people with autism have difficulty shifting attention and engage in inflexible and repetitive behaviors (Luna et al., 2002; Thakkar et al., 2008; Zikopoulos and Barbas, 2010). Among the areas that appear to be most affected by reduced functional connectivity are the right posterior inferior temporal gyrus, fusiform gyrus and bilateral inferior occipital cortex, which are related to facial recognition, a function often affected in ASD (Long et al., 2016).

Losses in S/N discrimination could also explain the alterations in auditory processing in ASD. Post-mortem studies have revealed that the density of dendritic spines in the temporal lobe is higher in ASDs than in TDs, showing a large age variability within the ASD group (Hutsler and Zhang, 2010). This variability, which probably depends on internal noise levels, both within and between individuals, could be representative of the process of synaptic pruning that relies on prior neuronal migration patterns.

In addition, individuals with ASD are often prone to hyper- or hypo-sensitivity to sound, light and texture, both of which fluctuate from birth to adolescence (Park et al., 2017). This is likely to be reflected in differences in speech-VS-noise discrimination in people with ASD, who are often able to show superior performance in simple psychoacoustic tasks, such as pitch memory and discrimination tasks, while being poor in tasks that require complex auditory processing, such as speech intonation, prosody, and visual-auditory integration (O’Connor, 2012; Dunlop et al., 2016).

Furthermore, ASD-affected individuals exhibit autonomic nervous system (ANS) functioning abnormalities, a common neuro-anatomical alteration underlying ASD. Over-activation of the sympathetic branch of the ANS due to ASD-specific parasympathetic activity deficit, may in turn induce deregulation of the gut-brain axis, thus attenuating intestinal immune and osmotic homeostasis (Beopoulos et al., 2021). The reason for this could be understood in terms of the different connectivity of the two branches of the ANS to the CNS.

The main inputs to the parasympathetic system, which exhibits activity deficit in ASD, originate mainly from limbic forebrain and cortical structures (orbital frontal gyri, cingulate cortex, thalamus, hypothalamus, amygdala, hippocampus). In this system, inputs from nerve X (vagus) connect to cardiac and smooth muscles, to the gastrointestinal tract and to secretory glands in lungs and viscera. In contrast, the main inputs to the sympathetic system, which doesn’t exhibit any activity deficit in ASD, originate from thoracic (vertebrae T1-L2) and sacral (vertebrae T1-L2) spinal cord. In this system, inputs from the sacral components connect, via the pelvic nerves, to smooth muscles, secretory glands in the lower gastrointestinal tract and to pelvic viscera.

Dysregulation of post-natal synaptic pruning and the ensuing losses of signal-over-noise discrimination are thus likely to affect the parasympathetic branch more directly and profoundly than the sympathetic branch. This is exemplified in pathological settings such as acute coronary artery disease, where psychological and bio-behavioral factors during the course of daily lives can provoke acute coronary events in individuals who do not fit the traditional high-risk profile for coronary artery disease (Ruberman et al., 1984). During mental stress, subcortical regions related with memory, emotion, and neuro-hormonal sympathetic control are activated. Instead, the frontal evaluative regions, such as the dorsal lateral prefrontal cortex associated with parasympathetic tone, are mostly deactivated.

Here, mental stress-mediated activation of visceral effectors from the CNS promotes parasympathetic withdrawal (HRV) and accentuation of sympathetic tone (norepinephrine) (Soufer et al., 2009) with subsequent β-adrenergic activation maintaining the autonomic imbalance (Brindle et al., 2014).

### The autonomic nervous system and gut homeostasis

The connectivity between the ANS and the immune system play important roles in controlling homeostasis and immune function of the gut. The sympathetic innervation secretes neurotransmitters that influence immune cells and the inflammatory response upon vagus nerve stimulation. By fine-tuning the interaction between the ENS and mucosal immune cells, the gut microbiota plays a role in the response (Bernardazzi et al., 2016; Beopoulos et al., 2021). ANS functioning abnormalities observed in association with ASD (sympathetic over-activation on a background of parasympathetic activity deficits) are very likely to affect neuro-immune interactions, resulting in the decreased secretion of bactericidal peptides and metabolites from Paneth cells, goblet cells and intestinal mucosal surfaces phagocytes, such as macrophages, which coexist with microbial communities (Peck et al., 2017), via an attenuation of autonomically mediated (Furness, 2016) stimulation of exosomal secretion (Fleshner and Crane, 2017) from colonic mucosa (Xu et al., 2016; Willemze et al., 2019), perhaps together with decreased lectins secretion (Pang et al., 2016). This stands to favor the loss of commensal microbial populations while concurrently dysregulating luminal ionic and water homeostasis, thus resulting in the highly heterogeneous and polymorphic dysbiosis concurrently with the unusually high incidences of gastrointestinal dysfunctions observed in association with ASD (Sgritta et al., 2019). These ANS functioning abnormalities apparently develop as a direct consequence of the dysregulations in early brain development and neuroglial migration patterns described above. It could then be maintained by the ensuing losses of signal-over-noise discrimination and subsequent chronic mental stress, as strongly suggested by the typically mental stress -associated comorbidities most frequently associated with autism (anxiety and depressive disorders, sleep problems, etc.). Furthermore, these ANS functioning abnormalities also adequately explain the origins of ASD-associated dysbiosis and the reasons for its persistence through a self-sustaining vicious circle whereby dysbiosis maintains dysregulated gut homeostasis which, in turn, maintains ANS functioning abnormalities which, in turn, maintains dysbiosis (Beopoulos et al., 2021).

It is important to note that defective synaptic pruning is also a hallmark of other neurodevelopmental disorders, although with different outcomes. Excessive pruning in all brain regions is associated with a loss of gray matter volume and is mainly related to schizophrenia, while extensive pruning in certain brain regions is associated with bipolar disorder. In contrast, ASD is mainly associated with defective pruning and increased white and gray matter volume, at least until adolescence. This could indicate differential patterns of neuronal migration occurring in different developmental windows during gestation, which could also depend on sudden changes in environmental conditions, inflammation and RNA editing. It is important to note that while schizophrenia and bipolar disorder are linked to low-grade systemic inflammation, there is no conclusive evidence for systemic inflammation in ASD (Dipasquale et al., 2017; Rosenblat and McIntyre, 2017; Osimo et al., 2018).

## Discussion/conclusion

The model presented here is rooted in very early neurogenesis and neuronal migration and, while it does not pretend to incorporate all the likely causes of ASD, it does highlight the conditions and developmental window where alterations in neural development can have major effects on a wide range of symptoms.

Neuronal migration patterns during cerebral cortex formation are largely governed by a combination of genetic, epigenetic and early environmental conditions that may well explain the focal disruption of cortical laminar architecture observed in ASD. The early presence of 5-HT in the brain, as well as its heterologous uptake throughout critical developmental periods, suggest its importance during early steps of brain development and wiring. Maternal low grade systemic inflammation particularly during early and mid-pregnancy, appears to be a major determinant for significantly increased risk of ASD pathogenesis. Sudden changes in environmental conditions, such as an over-supply of serotonin and/or kynurenine to the developing fetus, subsequently to a sustained low-level maternal inflammatory reaction, during a critical developmental window starting between GW8 and GW9 and probably extending to GW20, are likely to promote ASDs pathogenesis during fetal brain development. However, fluctuations in 5-HT are not sufficient to explain the prevalence of ASD, and individual susceptibility is therefore better explained by RNA editing mechanisms than by epigenetic mechanisms that require stable environmental conditions to act. Therefore, in order to provoke the cortical network disorganizations which will give rise to ASD pathogenesis, changes in central 5-HT levels during developmentally sensitive periods of brain development will probably have to occur in conditions where fetal genotypes, such as the S allele for the SLC6A4 gene in combination with CPEB4 and/or ADARs allelic variants would favor vulnerability over plasticity outcomes.

Postnatally, dysregulation of synaptic pruning as a result of neuronal migration patterns alterations during the key stages of cerebral cortex early development (between WP10 and WP30) stands to result in a huge variety of forms and degrees of signal-over-noise discrimination losses, thereby accounting for the enormous clinical heterogeneities encountered in autism, including ANS-associated functioning abnormalities. These are characterized by an over-activation of the sympathetic branch on a background of the parasympathetic activity deficits, which in turn, induces the deregulation of the gut-brain axis resulting in the maintenance of a persistent dysbiotic state that, in turn can negatively influence the gut-brain axis coordination. These phenomena may very well explain the enormous genetic and symptomatic heterogeneities that are systematically associated with ASD as well as the comorbidities most frequently associated with the pathology (anxiety and depressive disorders, sleep problems, gastrointestinal dysfunctions, etc.).

## Data availability statement

The original contributions presented in this study are included in the article/supplementary material, further inquiries can be directed to the corresponding author.

## Author contributions

FI: conceptualization and methodology. MG: software and project administration. FI, AB, MG, and AF: validation and resources. FI and AB: formal analysis, investigation, data curation, and writing—original draft preparation. FI, AB, and AF: writing—review and editing. All authors read and agreed to the published version of the manuscript.

## Footnotes

1
https://databases.lovd.nl/shared/variants/ADAR
